# Influence of prior alkalosis or acidosis on physiological responses during passive hyperthermia

**DOI:** 10.1113/EP092785

**Published:** 2026-02-27

**Authors:** Jacky Soo, Shernise Ng, Chris Abbiss, Olivier Girard, Mohammed Ihsan

**Affiliations:** ^1^ Discipline of Exercise Science Murdoch University Perth Western Australia Australia; ^2^ Sports Physiology Department, Sport Science and Medicine Centre Singapore Sports Institute Singapore Singapore; ^3^ Department of Physiology National University of Singapore Singapore Singapore; ^4^ School of Medical and Health Sciences Edith Cowan University Joondalup Western Australia Australia; ^5^ School of Human Sciences (Exercise and Sport Science) The University of Western Australia Crawley Western Australia Australia; ^6^ Physical Education Department, College of Education United Arab Emirates University Al Ain UAE

**Keywords:** acidosis, alkalosis, hyperthermia, thermoregulation

## Abstract

This study manipulated the blood pH of participants to be mildly acidic or basic prior to passive hyperthermia to examine its effects on thermoregulatory and chemo‐regulatory responses, including ventilatory parameters, rectal temperature (*T*_re_) and thermal perception. Twelve healthy males attended three experimental sessions in a double‐blind, randomised, counterbalanced design. Passive hyperthermia was induced by hot water immersion at 40°C following the consumption of corn starch (placebo; PLA), ammonium chloride (AC) or sodium bicarbonate (SB). Compared with PLA, SB consumption increased blood pH and HCO_3_^−^, whilst AC decreased both blood pH and HCO_3_^−^ (all *P *< 0.001). Specifically, minute ventilation was unchanged following SB (11.0 ± 3.7 L min^−1^) but higher following AC (13.2 ± 5.3 L min^−1^) compared with PLA (11.9 ± 5.1 L min^−1^; *P *= 0.002). Changes in ventilatory equivalent for O_2_ and CO_2_ were greater in AC and lower in SB compared with PLA (*P *< 0.05). *T*_re_ increased similarly across all conditions (*P *= 0.089), whilst immersion times approached a difference (SB: 51.1 ± 10.2 min, AC: 52.9 ± 8.1 min, and PLA: 56.8 ± 6.8 min; *P *= 0.06). Thermal sensation was higher in AC compared with PLA and SB (all *P *< 0.001), with no difference between PLA and SB (*P *= 0.45). Thermal discomfort was not different between AC and SB (*P *= 0.66), both of which were higher than PLA (all *P *< 0.001). The magnitude and characteristics of ventilatory response during hyperthermia were influenced by prior alkalemia or acidosis, coinciding with differences in thermal perceptions.

## Abstract (highlights)

- **What is the central question of this study?**
  How do acidosis and alkalosis influence ventilatory response (i.e., minute ventilation), core temperature and thermal perception during passive hyperthermia?
- **What is the main finding and its importance?**
  Prior alkalosis did not decrease absolute ${\dot{V}}_{E}$ during passive hyperthermia but decreased ventilatory equivalents, indicating a relative decrease in ${\dot{V}}_{E}$ relative to CO_2_ removal. Prior acidosis increased ventilation during hyperthermia. Our findings indicate chemoreceptor activity in modulating acid–base balance and ventilatory responses during hyperthermia, which are likely dependent on the degree of hyperthermia and the initial pH state.

## 1 INTRODUCTION

Passive (Fujii et al., 2008b; Nelson et al., 2011) and exercise‐induced hyperthermia (Beaudin et al., 2009; Tsuji et al., 2012) have been shown to increase ventilation in humans (i.e., thermal hyperpnoea). This increased ventilatory drive—the intensity of the neural output from the respiratory centre regulating ventilatory responses— enhances CO_2_ removal from the lungs compared with that created by the tissues, leading to a decrease in arterial CO_2_ partial pressure ($P_{\mathrm{aC}O_{2}}$) (hypocapnia) (Gaudio & Abramson, 1968; Tsuji et al., 2016), and an increase in arterial pH (alkalosis) (Abbiss et al., 2007; González‐Alonso et al., 1998).

Whilst the physiological significance of thermal hyperpnoea in humans remains unclear (Tsuji et al., 2016), this phenomenon seems strongly linked to mechanisms maintaining heat balance. Some studies (Crandall et al., 2011; White et al., 2011) have postulated that this response may facilitate selective brain cooling (Cabanac, 1993; Nagasaka et al., 1998) or enhance heat dissipation through respiratory heat loss (Hanson, 1974), though this is not universally agreed upon (Crandall et al., 2011; Nybo & Secher, 2011). Others have shown that ensuing hypocapnia can induce cerebral hypoperfusion (Nelson et al., 2011), which may impede cerebral heat exchange (Tsuji et al., 2016). A decrease in ventilatory drive is sometimes observed following hyperpnoea‐induced hypocapnia, likely as a mechanism to limit excessive decreases in $P_{\mathrm{aC}O_{2}}$ and hence maintain acid–base homeostasis (Hayashi et al., 2011; Tsuji et al., 2016). Together, these findings highlight the importance of the ventilatory response in regulating acid–base balance and possibly core temperature.

In contrast, Abbiss et al. (2007) showed that during prolonged self‐paced exercise in the heat, hyperthermic‐induced hyperventilation remained elevated, despite pH increasing to near alkalaemia (7.50 ± 0.04). To mitigate heat‐induced hyperventilation and associated cerebral hypoperfusion, Katagiri et al. (2021) proposed the use of sodium bicarbonate (SB) ingestion, which reduced minute ventilation (${\dot{V}}_{E}$), which in turn attenuated the decrease in $P_{\mathrm{aC}O_{2}}$; this limited cerebral blood flow decreases during prolonged submaximal exercise in the heat, without altering ventilatory sensitivity or the core temperature threshold for hyperventilation. However, in resting humans exposed to passive heating, SB ingestion resulted in similar ventilatory responses, ventilatory sensitivity and cerebral blood flow compared with control (Katagiri et al., 2024). Collectively, these findings suggest exercise‐ and rest‐specific effects of SB ingestion, supporting its role in mitigating hyperthermia‐induced hyperventilation and cerebral hypoperfusion during exercise, but not rest. By extension, pH‐sensitive chemoreceptor activity may not be a primary driver of hyperthermia‐induced hyperventilation during passive heating.

The passive heating protocol (Katagiri et al., 2024) provides a useful model for isolating pH‐related influences (induced during exercise) on ventilatory regulation, by removing mechanical and metabolic factors (e.g., H^+^ ions). Yet, a comprehensive understanding requires a bidirectional approach. Specifically, the lack of an alkalosis effect on ventilatory response does not preclude an acidosis effect, which may better elucidate chemoreceptor involvement. Indeed, ingestion of ammonium chloride (AC) induces metabolic acidosis and can elevate ventilation (Kowalchuk et al., 1984; Tojima et al., 1986), though its effect on hyperthermia‐induced hyperventilation remains unclear. Furthermore, the functional implications of acid–base manipulation for heat tolerance and perceptual responses are unknown. It is plausible that ventilatory changes and the consequent influence on cerebrovascular perfusion may ultimately affect time to volitional intolerance in the heat.

The aim of this study is to examine the thermoregulatory and chemo‐regulatory responses, including ventilatory response, core temperature (*T*_c_), and thermal perception during passive hyperthermia by manipulating initial pH to be either mildly acidic or basic. It is hypothesised that prior alkalosis will not alter hyperthermia‐induced hyperventilation beyond that observed under control, whereas prior acidosis may exacerbate hyperventilation and consequently hypocapnia. Additionally, we hypothesise that these ventilatory adjustments would influence thermal perception and heat tolerance during passive heating.

## 2 METHODS

### 2.1 Ethical approval

This study was approved by the Institutional Review Board of the Singapore Sports Institute (PH‐FULL‐014). All participants were informed of the study requirements, and written informed consent was obtained prior to participation. This study was performed in accordance with the *Declaration of Helsinki*, except for registration in a database.

### 2.2 Participants

Using ${\dot{V}}_{E}$ as the primary outcome measure, an a priori power analysis revealed that a sample size of six participants would provide sufficient power (0.80), accounting for type 1 error (0.05) and expected change in ${\dot{V}}_{E}$ (effect size, *d* = 1.5) (Katagiri et al., 2024; Tsuji et al., 2019). Twelve healthy, physically active males (age: 24.5 ± 1.7, height: 175.4 ± 4.6 cm and body weight: 72.1 ± 9.8 kg) were recruited for this study. All participants had no history of cardiovascular or respiratory diseases or prior heat injury.

### 2.3 Experimental overview

Participants attended three experimental sessions, prior to which they abstained from exercise, caffeine and alcohol for 24 h. Passive hyperthermia was administered via hot water immersion (HWI) following the consumption of either corn starch (placebo; PLA), ammonium chloride (AC) or sodium bicarbonate (SB) to induce acidosis and alkalosis, respectively. The experimental trials were conducted at the same time of the day, separated by at least 5–7 days, in a double‐blind, randomised, counterbalanced and crossover design.

### 2.4 Experimental procedure

#### 2.4.1 Acid–base manipulation

Participants were asked to consume their regular meal 2–3 h before arriving at the laboratory (4–5 h before HWI treatment). This was to minimise possible side effects from ingesting acid or alkali‐based supplements (Siegler et al., 2010). Upon arrival at the laboratory, participants were provided with a beverage consisting of 0.3 g kg^−1^ body weight (BW) of 60% maltodextrin and 40% protein (whey) 150 min prior to HWI (Siegler et al., 2015). Following 1 h of rest, they then started consuming the PLA, AC or SB (0.3 g kg^−1^ BW packed in gelatin capsules). The supplements were equally divided into three doses and consumed at 90, 60 and 30 min before HWI treatment, along with a total of 1 L of water. The AC and SB dosages (i.e., 0.3 g kg^−1^ BW) were based on studies showing adequate tolerance (Ducker et al., 2013; Hollidge‐Horvat et al., 2000) as well as significant pH changes (pH of 7.25–7.35 for AC and 7.43–7.49 for SB) (Siegler et al., 2015).

#### 2.4.2 Hot water immersion treatment

All trials were conducted in standard laboratory conditions (23.6 ± 1.1°C and 71.1 ± 5.0% RH). Baseline (0) ventilatory measurements (Parvomedics Trueone 2400, Sandy, USA) were taken 25 min after the last dose for 5 min. Participants were then immersed in hot water (up to the level of the mid‐sternum) in a semi‐reclined position (IC‐iBody, IC‐Heat, Australia). The water temperature was maintained at 40°C with a heating pump unit (IC‐Heat, iCoolsport, Gold Coast, Australia). During HWI, participants reported ratings of thermal sensation (13‐point scale) (Lee et al., 2010) and thermal discomfort (10‐point scale) (Griffiths & Boyce, 1971) at 5‐min intervals. The HWI was terminated when any of the following three conditions were met: (1) a rectal temperature (*T*_re_) threshold of 39.5°C, (2) the 60‐min time limit, or (3) volitional termination when participants rated 13 or 10 on the thermal sensation and thermal discomfort scale, respectively. The 60‐min time limit was based on previous studies reporting immersion times of 44.9 ± 12.9 min (Périard et al., 2011) or 40–50 min (Sabapathy et al., 2021) for *T*_re_ to reach at least 39.5°C in water baths maintained at 40°C or 41–42°C, respectively.

### 2.5 Measurements

#### 2.5.1 Blood sampling

Capillary blood samples were collected from the participants’ (*n* = 6) index fingers using a 125 µL heparinised capillary tube (Clinitubes, Radiometer, Copenhagen, Denmark) and analysed for blood pH and blood bicarbonate (HCO_3_^−^) using the iStat Blood Gas Analyser (i‐STAT Corporation, East Windsor, NJ, USA). Blood sampling and analysis were undertaken 90 min before HWI treatment (before the first dose: Rest), immediately before HWI treatment (0), at 10 min intervals during the HWI, and following termination of HWI (End).

#### 2.5.2 Core temperature

*T*_re_ was measured via disposable probes (MEAS 4400 Temperature Probe, Measurement Specialities Inc., PA, USA), which participants self‐inserted to a depth of 12 cm past the anal sphincter. The probe was connected to a data logger (Cole Parmer Thermistor Thermometer 8502‐12, Cole Parmer Instrument Co., IL, USA), and *T*_re_ was recorded every 5 min throughout HWI.

#### 2.5.3 Heart rate and ventilatory responses

Heart rate (HR) was monitored (Polar RS400, Polar Electro, Kempele, Finland) every 5 min throughout HWI. At the 3.5‐min mark of each 5‐min interval during HWI, participants were fitted with a mouthpiece, and expired gases were sampled for 90 s via an automated system (TrueOne, Parvomedics). Respiratory measurements from the last 30 s of each sampling period were retrospectively retrieved and averaged to represent a single value for each 5‐min time point. Derived variables included minute ventilation (${\dot{V}}_{E}$), respiratory rate, tidal volume (*V*_T_) and ventilatory equivalents for O_2_ (${\dot{V}}_{E}/{\dot{V}}_{O_{2}}$) and CO_2_ (${\dot{V}}_{E}/{\dot{V}}_{CO_{2}}$).

### 2.6 Data and statistical analysis

Immersion times between conditions (PLA, AC and SB) were analysed using one‐way repeated measures analysis of variance (ANOVA). Time‐dependent changes in ventilatory responses (${\dot{V}}_{CO_{2}}$, ${\dot{V}}_{E}$, ${\dot{V}}_{E}/{\dot{V}}_{O_{2}}$ and ${\dot{V}}_{E}/{\dot{V}}_{CO_{2}}$), HR, *T*_re_, thermal sensation, and thermal discomfort were analysed using linear‐mixed method modelling between condition (Condition [PLA, AC, and SB] × Time [0–60 min, at 5 min intervals]). Condition and time were included as fixed effects, and participants were treated as random effects. Similarly, time‐dependent changes in blood variables (i.e., pH and HCO_3_^−^) were analysed using a linear mixed method modelling with fixed effects for condition (PLA, AC and SB) and time (Rest, 0–60 min, at 10 min intervals). Temperature‐dependent changes in ventilatory responses and HR were examined using a linear mixed‐method model with fixed effects being defined as condition (PLA, AC and SB) and temperature (Rest, 37.5, 38.0, 38.5°C and End). When sampling intervals did not align with the *T*_re_ points, respiratory variables were estimated using linear interpolation. Significant main effects or interactions were analysed using Fisher's LSD *post hoc* analysis. Effect size estimates (*g*) were calculated and interpreted as small (*g* = 0.2), moderate (*g* = 0.5) or large (*g* = 0.8). Statistical calculations were performed using SPSS statistical software (SPSS version 19, IBM Corp., Armonk, NY, USA) and variables were deemed significant when *P* ≤ 0.05. All data was presented as means ± standard deviation (SD).

## 3 RESULTS

### 3.1 Immersion time and blood variables

**FIGURE 1:**
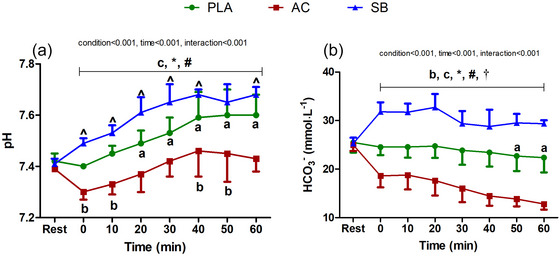
Time‐dependent changes in pH (a) and blood bicarbonate (b) during hot water immersion following placebo (PLA), ammonium chloride (AC) or sodium bicarbonate (SB) administration. a, b and c denote significant differences compared with baseline within PLA, AC and SB, respectively. * represents a significant difference between AC and SB; # represents a significant difference between PLA and AC; and † represents a significant difference between PLA and SB. *n* = 6 for all time points.

Immersion times between PLA (56.8 ± 6.8 min), AC (52.9 ± 8.1 min) and SB (51.1 ± 10.2 min) approached significance (*P* = 0.062). Changes in pH and blood HCO_3_^−^ are presented in Figure 1. Blood pH was significantly lower in AC (7.30 ± 0.03; *P* = 0.02, *g* = 3.65) and higher in SB (7.41 ± 0.02; *P* < 0.001; *g *= 3.93) prior to HWI compared with rest (before ingestion of the first dose, AC: 7.39 ± 0.01; SB: 7.41 ± 0.02). Compared with PLA, blood pH during the HWI was significantly lower in AC (all *P* ≤ 0.001; *g* = 1.27–4.20) from 0–60 min, and higher in SB from 0–40 min and at 60 min (all *P* ≤ 0.01, *g* = 0.58–4.83).

The ingestion of the supplements significantly altered blood HCO_3_^−^ concentrations. HCO_3_^−^ concentrations were significantly higher in SB (31.8 ± 1.9 mmol L^−1^; *P* < 0.001, *g* = 3.74) and lower in AC (18.6 ± 2.4 mmol L^−1^; *P* < 0.001, *g* = 3.09) at the start of the HWI treatment (time point 0) compared with rest (before ingestion of the first dose, AC: 25.0 ± 1.2 mmol L^−1^; SB: 25.3 ± 1.2 mmol L^−1^). Compared with PLA, HCO_3_^−^ concentrations were significantly higher in SB (*P* < 0.001, *g* = 3.72) and lower in AC (*P* < 0.001, *g* = 2.66) at the start of the HWI treatment (time point 0). Blood HCO_3_^−^ concentration remained consistently elevated in SB (all *P* < 0.001; *g* = 1.54–3.72) and reduced in AC (all *P* < 0.001; *g* = 2.06–3.30) throughout all time points during HWI.

### 3.2 Ventilatory parameters

**FIGURE 2:**
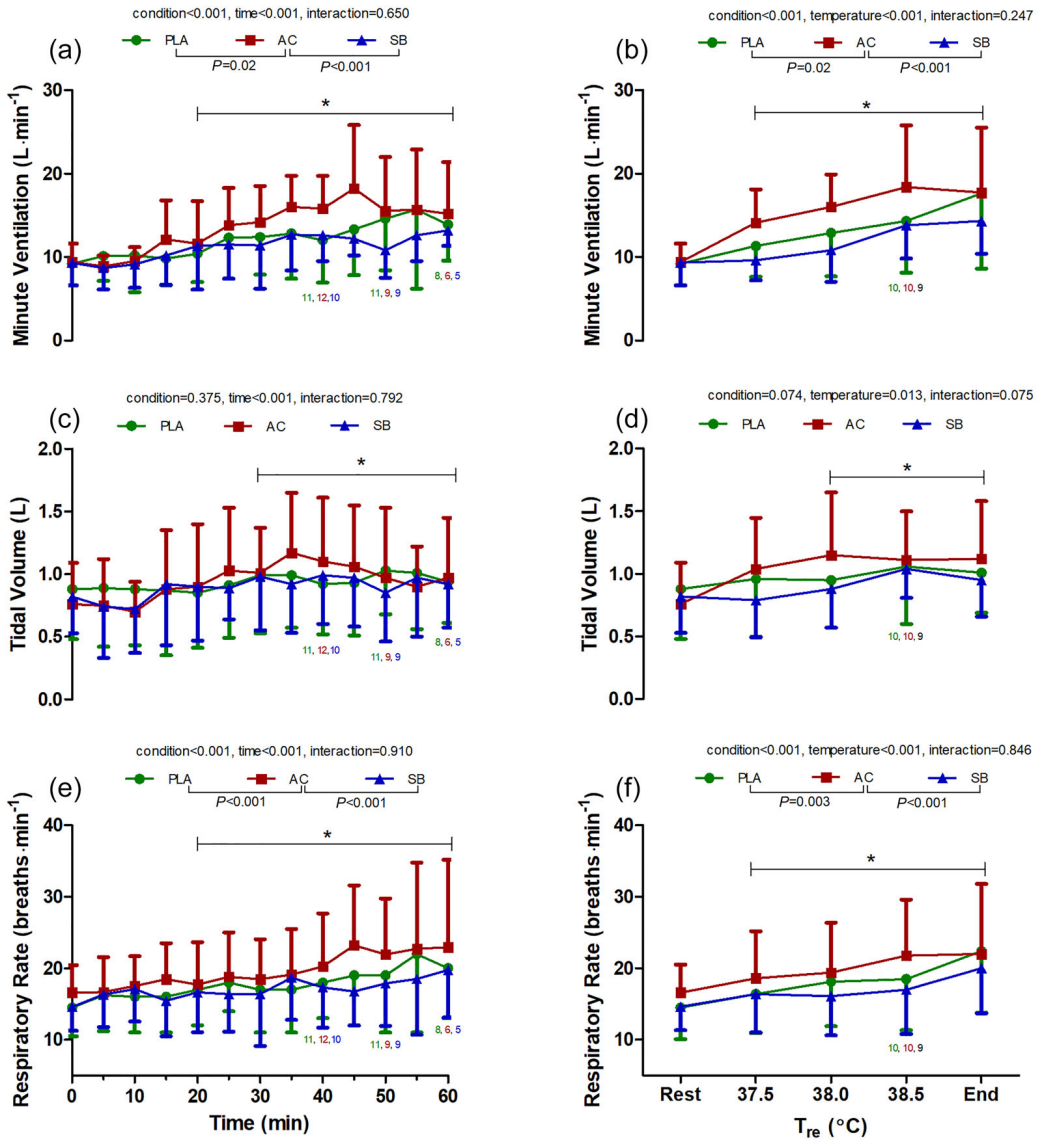
Time‐ and temperature‐dependent changes in minute ventilation (a, b), tidal volume (c, d), and respiratory rate (e, f) during hot water immersion following placebo (PLA), ammonium chloride (AC) or sodium bicarbonate (SB) administration. * represents post‐comparison for time effect regardless of condition. *Post hoc* comparisons between conditions are denoted in the legend. *n* = 12 unless indicated by coloured numbers corresponding to figure legend at specific time/temperature points.

**FIGURE 3:**
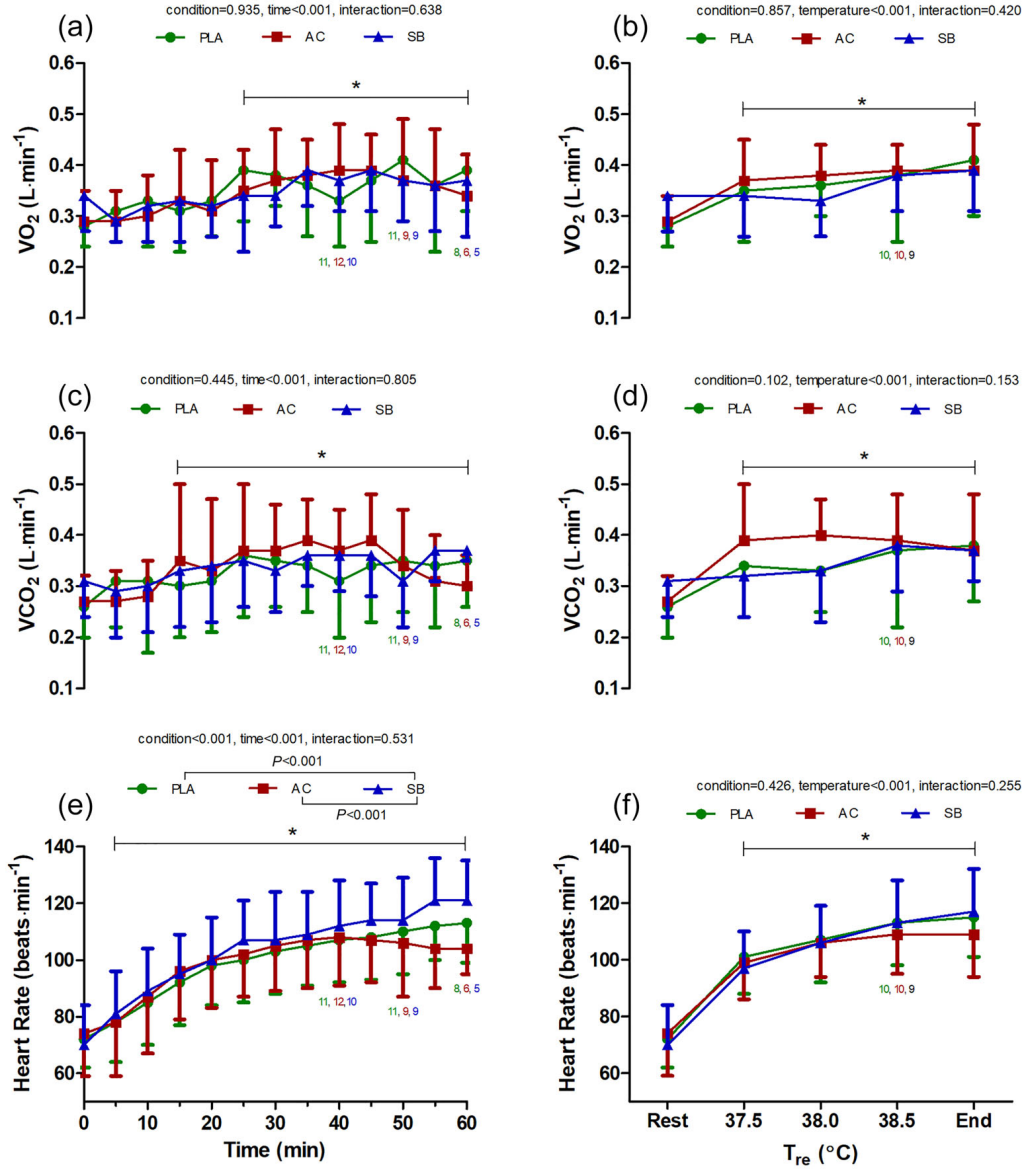
Time and temperature‐dependent changes in ${\dot{V}}_{O_{2}}$ (a, b), ${\dot{V}}_{CO_{2}}$ (c, d) and heart rate (e, f) during hot water immersion following placebo (PLA), ammonium chloride (AC) or sodium bicarbonate (SB) administration. * represents post‐comparison for time effect regardless of condition. *Post hoc* comparisons between conditions are denoted in the legend. *n* = 12 unless indicated by coloured numbers corresponding to figure legend at specific time/temperature points.

**FIGURE 4:**
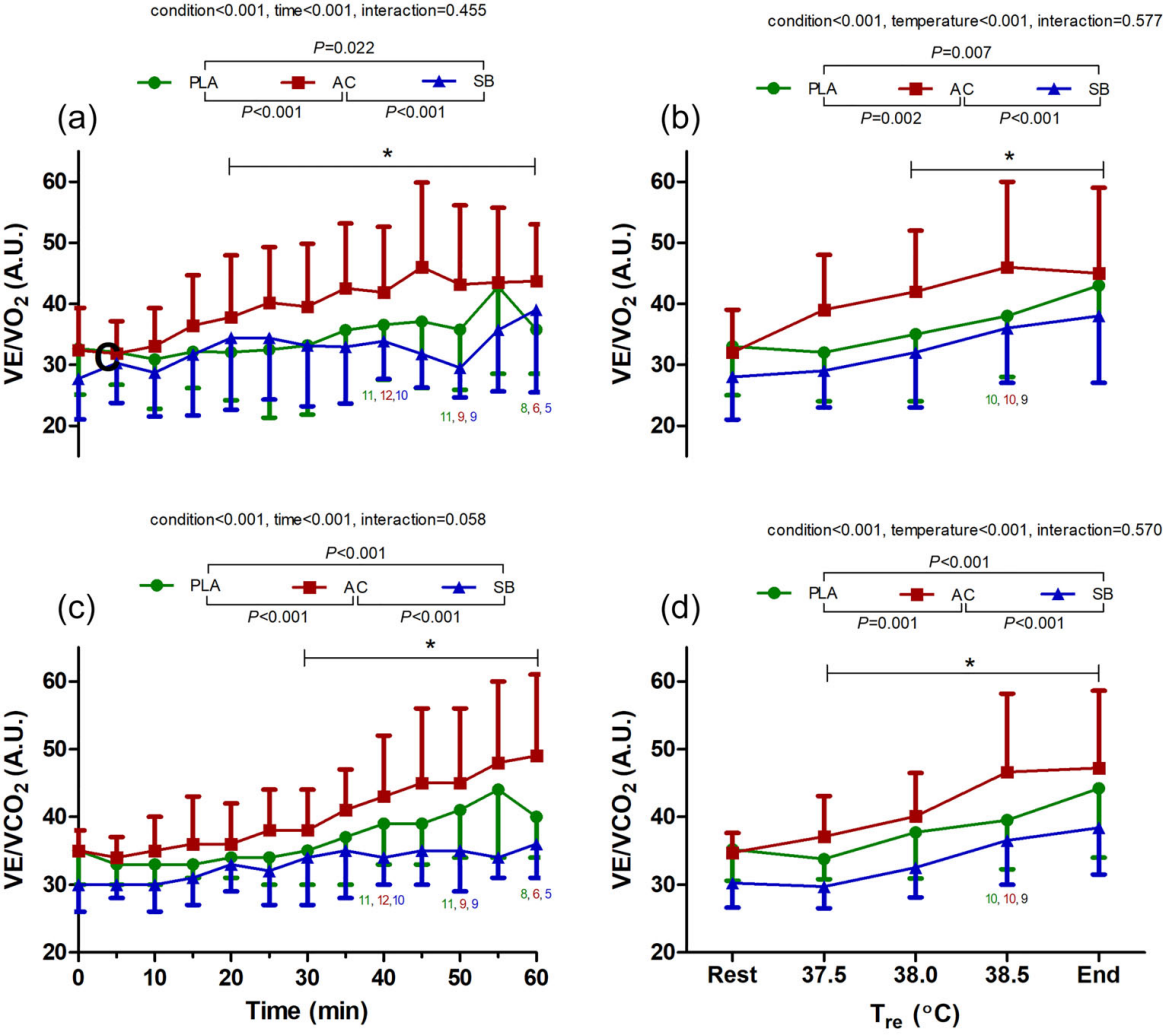
Time and temperature‐dependent changes in ${\dot{V}}_{E}/{\dot{V}}_{O_{2}}$ (a, b) and ${\dot{V}}_{E}/{\dot{V}}_{CO_{2}}$ (c, d) during hot water immersion following placebo (PLA), ammonium chloride (AC) or sodium bicarbonate (SB) administration. * represents post‐comparison for time effect regardless of condition. *Post hoc* comparisons between conditions are denoted in the legend. *n* = 12 unless indicated by coloured numbers corresponding to figure legend at specific time/temperature points.

Time‐ and temperature‐dependent changes in ventilatory parameters are shown in Figures 2, 3, 4. Both time‐ and temperature‐dependent changes in ${\dot{V}}_{E}$ (Figure 2a, b) showed significant main effects for condition and time/temperature. Specifically, time‐dependent changes in ${\dot{V}}_{E}$ were significantly higher in AC (pooled average from 0–60 min: 13.2 ± 5.3 L min^−1^) compared with PLA (11.9 ± 5.1 L min^−1^; *P* = 0.002; *g* = 0.24) and SB (11.0 ± 3.7 L min^−1^; *P* < 0.001; *g* = 0.48). However, changes in time‐ and temperature‐dependent ${\dot{V}}_{E}$ were not significantly different between PLA and SB (*P* = 0.09 and 0.10; *g* = 0.20 and 0.29). ${\dot{V}}_{CO_{2}}$ increased with time (*P* < 0.001) and temperature (*P* < 0.001) but did not demonstrate significant condition or interaction effects (Figure 3c, d).

Both ${\dot{V}}_{E}/{\dot{V}}_{O_{2}}$ and ${\dot{V}}_{E}/{\dot{V}}_{CO_{2}}$ increased over time and with temperature (all *P* < 0.001; Figure 4a–d). Specifically, changes in ${\dot{V}}_{E}/{\dot{V}}_{O_{2}}$ and ${\dot{V}}_{E}/{\dot{V}}_{CO_{2}}$ were significantly greater in AC compared with PLA and SB (all *P* ≤ 0.002; *g* = 0.33–0.94). Additionally, changes in ${\dot{V}}_{E}/{\dot{V}}_{O_{2}}$ and ${\dot{V}}_{E}/{\dot{V}}_{CO_{2}}$ were significantly lower in SB compared with PLA (*P* < 0.001–0.02; *g* = 0.24–0.67).

Respiratory rates increased with time and temperature, with larger increases in AC (time dependent pooled average: 19.2 ± 6.8 breaths min^−1^; temperature dependent: 19.6 ± 7.3 min^−1^) compared with PLA (time dependent: 17.5 ± 6.2 breaths min^−1^, *P* < 0.001, *g* = 0.25; temperature dependent: 18.0 ± 6.8 breaths min^−1^, *P* = 0.003; *g* = 0.22), whilst respiratory rates changes were similar between PLA and SB (*P* = 0.13 and 0.29; *g* = 0.12 and 0.18). Tidal volume increased with time and temperature but displayed no significant condition or interaction effects (Figure 2a, d).

### 3.3 Heart rate, rectal temperature and thermal perception

**FIGURE 5:**
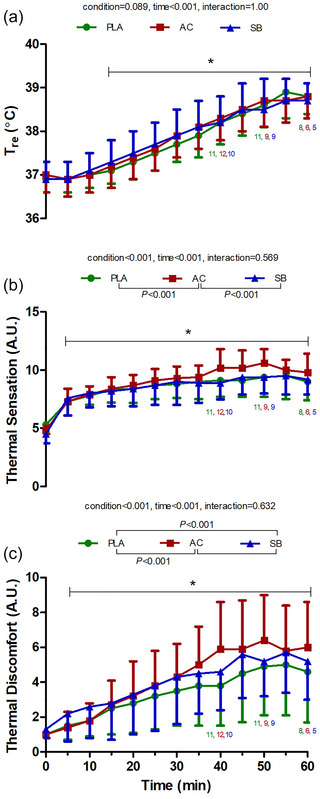
Time‐dependent changes in rectal temperature (a), thermal sensation (b) and thermal discomfort (c) during hot water immersion following placebo (PLA), ammonium chloride (AC) or sodium bicarbonate (SB) administration. * represents post‐comparison for time effect regardless of condition. *Post hoc* comparisons between conditions are denoted in the legend. *n* = 12 unless indicated by coloured numbers corresponding to figure legend at specific time/temperature points.

Heart rate increased during HWI (Figures 3e, f) with time in all conditions. Time‐dependent increases in HR were higher in SB (pooled average: 101 ± 20 bpm) compared with AC (97 ± 20 bpm; *P* < 0.001, *g* = 0.14) and PLA (98 ± 19 bpm; *P* < 0.001, *g* = 0.17); changes in HR were not significantly different between PLA and AC (*P* = 0.46, *g* = 0.04). *T*_re_ increased with time in all conditions (*P* < 0.001), with no significant difference between conditions (*P* = 0.09). Thermal perceptions, including thermal sensation and thermal discomfort, increased with time in all conditions (all *P* < 0.001; Figure 5b, c). Increases in thermal sensation were higher in AC compared with PLA and SB (all *P* < 0.001, *g* = 0.24 and 0.25), but did not differ between PLA and SB (*P* = 0.446, *g* = 0.02). Thermal discomfort was significantly higher in AC and SB compared with PLA (all *P* < 0.001, *g* = 0.26 and 0.20); no significant difference in thermal discomfort was observed between AC and SB (*P* = 0.659, *g* = 0.07).

## 4 DISCUSSION

This study examined the thermoregulatory and chemo‐regulatory responses, including ventilatory responses, *T*_re_, and thermal perception during passive hyperthermia by selectively manipulating initial pH to be either mildly acidic or basic. As expected, prior alkalosis did not alter hyperthermia‐induced hyperventilation beyond that observed under control but decreased ventilatory equivalents, indicating a relative decrease in ${\dot{V}}_{E}$ relative to CO_2_ removal. In contrast, prior acidosis resulted in higher hyperventilation. Contrary to our hypothesis, despite higher ventilatory response and thermal perceptions in AC, heat tolerance was not altered beyond that observed in PLA.

### 4.1 Efficacy of supplementation on blood acid–base balance

The dosages of the SB and AC supplements demonstrated adequate tolerance (Ducker et al., 2013; Hollidge‐Horvat et al., 2000) and significant pH changes (Siegler et al., 2015). As expected, SB and AC supplementation effectively altered the acid–base status of the participants, as evidenced by an increase in blood pH and HCO_3_^−^ in SB, and a decrease in blood pH and HCO_3_^−^ with AC (Figure 1). The magnitudes of these changes were comparable to previous work (Siegler et al., 2015).

### 4.2 Effect of sodium bicarbonate ingestion on ventilatory response

It was hypothesised that prior alkalosis would not influence ventilation. This hypothesis stemmed from recent work by Katagiri et al. (2024), who showed that SB consumption did not mitigate hyperthermia‐induced hyperventilation compared with a control condition involving sodium chloride ingestion. In agreement, time‐ and temperature‐dependent changes in ${\dot{V}}_{E}$ were not different following PLA and SB ingestion in this study. However, ${\dot{V}}_{E}/{\dot{V}}_{O_{2}}$ and ${\dot{V}}_{E}/{\dot{V}}_{CO_{2}}$ were significantly lower following SB ingestion (Figure 4). Given that no condition effects were observed with regard to ${\dot{V}}_{CO_{2}}$, these changes in the ventilatory equivalents indicate a decrease in ${\dot{V}}_{E}$ relative to CO_2_ elimination and O_2_ uptake in the lungs. Accordingly, this suggests that prior alkalosis may have suppressed chemoreceptor activity, which manifested not as a reduction in absolute ${\dot{V}}_{E}$, but as a relative measure with regard to CO_2_ elimination per unit of ventilation. Our results regarding absolute ${\dot{V}}_{E}$ and ventilatory equivalents align with the findings reported by Katagiri et al. (2024). This indicates that prior alkalosis does not alter the magnitude of the thermally driven ventilatory response during passive heating. Instead, it modifies the ventilatory characteristics to mitigate excessive decrease in CO_2_, likely through attenuated chemoreceptor activity. Given that SB consumption lowers plasma H^+^ and increases plasma HCO_3_^−^ concentrations (Lindinger et al., 1999), the suppressed ventilatory response may be partly due to the reduction in blood H^+^ caused by SB supplementation (Katagiri et al., 2021). However, given the passive heating model used in this study, this mechanism is less likely to be of significance, as metabolic perturbations are minimal compared with exercise and thus modestly account for the observed changes in ventilatory equivalents.

### 4.3 Effects of sodium bicarbonate ingestion on *T*_re_ and thermal perception

Similar increases in *T*_re_ were observed between SB and PLA throughout HWI (Figure 5). These findings are consistent with recent works showing that prior SB supplementation does not influence changes in oesophageal temperature (relative to placebo) in hyperthermic humans at rest and during exercise (Katagiri et al., 2024, 2021). These findings indicate that decreases in ventilation or its characteristics do not impact the rate of heat storage during both exercise (Katagiri et al., 2021) and passive hyperthermia challenges (Katagiri et al., 2024). However, thermal discomfort was higher in SB compared with PLA, despite the tendency for shorter immersion times occurring in SB (51.1 ± 10.2 min) compared with PLA (56.8 ± 6.8 min) (*P* = 0.06). Whilst speculative, this may be attributable to generalised discomfort (e.g., mild gastrointestinal distress) associated with SB ingestion, which was not directly assessed in the present study. Further research is required to dissociate the physiological and perceptual responses to hyperthermia under alkalotic conditions.

### 4.4 Effect of ammonium chloride on ventilatory response

By decreasing the pH prior to hyperthermia via AC supplementation, we aimed to examine the effects of reduced pH on ventilatory responses as well as investigate the chemoreceptor involvement during hyperthermia. In this regard, ventilatory responses were greater following AC supplementation compared to SB and PLA, as evidenced by higher ${\dot{V}}_{E}$ (Figure 2a, b), ${\dot{V}}_{E}/{\dot{V}}_{O_{2}}$ and ${\dot{V}}_{E}/{\dot{V}}_{CO_{2}}$ (Figure 4) across all time points. These effects were primarily mediated by increased respiratory rate, rather than tidal volume (Figure 2c–f). These findings are consistent with previous work demonstrating increased ventilatory responses after acidosis during rest (Tojima et al., 1986) and exercise (Kowalchuk et al., 1984), although these studies did not involve hyperthermia challenges. Thus, the present study provides novel data on the combined effects of AC and hyperthermia on ventilatory responses.

We expected that the dual challenges of prior acidosis and hyperthermia would lead to pronounced hypocapnia (i.e., decreased $P_{\mathrm{ETC}O_{2}}$), potentially triggering a curtailed hyperventilatory response (i.e., hypoventilatory drive) to maintain acid–base homeostasis balance. This is supported by previous studies (Hayashi et al., 2011; Tsuji et al., 2016) showing a decrease in ventilatory drive during exercise, as a consequence of hyperpnoea‐induced hypocapnia, likely as a mechanism to limit excessive decreases in $P_{\mathrm{aC}O_{2}}$. Contrary to this expectation, we did not observe a decrease in the hyperventilatory response following AC supplementation, compared with PLA (Figure 4). Similarly, Tsuji et al. (2018) reported that ${\dot{V}}_{E}$ and its sensitivity to core temperature were comparable between hypocapnic and eucapnic states in passively heated humans. Collectively, these findings highlight the distinction in ventilatory responses between passive and exercise heat stress (Hayashi et al., 2011; Tsuji et al., 2018, 2016). Specific to this study, it is plausible that the sustained hyperventilation reflected respiratory compensation (as a consequence of hypocapnia), effectively offsetting the imposed metabolic acidosis and restoring pH toward baseline.

Nonetheless, it should be highlighted that the effect of hypocapnia on ventilatory drive during hyperthermia remains inconclusive, with studies reporting unchanged (Fujii et al., 2008a), increased (Nelson et al., 2011), or decreased (Bain et al., 2013) ventilation after CO_2_ pressure restoration during passive heat stress. Importantly, the acidosis induced in this study was mild (i.e., 7.39 ± 0.01 to 7.30 ± 0.03 following 3 doses) and may not have reached the threshold required to trigger hypoventilation (Yagi & Fujii, 2021). Hence, within the limits of metabolic acidosis and hyperthermia imposed in the present study, our findings indicate that a persistent hyperventilatory response was maintained.

### 4.5 Effect of ammonium chloride on *T*_re_ and thermal perception

The increase in *T*_re_ was comparable between AC and PLA throughout HWI. However, immersion times tended to be shorter in AC (52.9 ± 8.1 min) compared with PLA (56.8 ± 6.8 min), albeit statistical significance was not reached (*P* = 0.06). In this context, the shorter immersion time in AC was accompanied by higher thermal sensation and discomfort. Although the exact reasons are not completely understood, we speculate that the sustained hyperventilatory drive following AC‐induced hypocapnia, which in turn might have exacerbated cerebral hypoperfusion (Nelson et al., 2011) and impaired heat exchange within the brain (Tsuji et al., 2016), resulting in greater thermal perception. It should be noted, however, that blood velocity, measured using transcranial Doppler, is typically used as a surrogate measurement of cerebral blood flow in previous studies (Nelson et al., 2011; Tsuji et al., 2018). As such, the effect of hypocapnia on cerebral blood flow remains unclear, and further objective data are needed to verify this suggestion.

### 4.6 Limitations and additional considerations

The AC and SB supplementation protocol used in this study was based on prior research demonstrating its effectiveness in altering acid–base status, as well as minimising gastrointestinal distress such as vomiting or diarrhoea. Whilst we did not observe any of these symptoms during all experimental sessions, we note that gastrointestinal tolerance was not directly assessed; as such, milder gastrointestinal issues may have been unreported. AC and SB are commonly used models of acidosis and alkalosis, respectively. However, the use of these supplements may also alter plasma osmolality and/or volume. In this regard, further research is required to determine if changes in plasma osmolality may alter the hyperthermic‐induced hyperventilatory response. Rectal temperature was used as a surrogate of core temperature in this study. However, oesophageal temperature was used by others (Hayashi et al., 2011; Katagiri et al., 2024) given its proximity to the ventricle, and its closer approximation to brain temperature (Whitby & Dunkin, 1971). That said, technical difficulties with inserting the probe and discomfort (e.g., irritation to the nasal passage) are common (Moran & Mendal, 2002). Regardless, whilst we acknowledge that rectal temperature may not reflect brain temperature, it remains a valid index for the monitoring of the temperature of the deep body tissue. Ideally, arterial blood samples should be used for the measurement of blood pH and HCO_3_^−^, although it is not commonly used due to the invasiveness and therefore ethical and safety concerns (e.g., arterial line for multiple samplings). Consequently, capillary blood sampling is routinely used in both research (Abbiss et al., 2007) and clinical settings (Yıldızdaş et al., 2004) and should correlate closely with arterial blood measurements (Yıldızdaş et al., 2004). In this regard, the acid–base status of our participants following supplementation is consistent with results from previous studies (Ducker et al., 2013; Hollidge‐Horvat et al., 2000; Siegler et al., 2015). Finally, the core temperature threshold for increases in ventilation and/or the sensitivity of ${\dot{V}}_{E}$ to increasing core temperature could not be determined in this study, as we were not able to measure ventilatory responses continuously. In particular, we observed that the participants experienced significant discomfort during the pilot testings when they were asked to breathe through a mouthpiece (with nose clipped) continuously throughout the trial; consequently, the test was prematurely terminated due to discomfort unrelated to the HWI. As such, ventilatory responses were only measured at 5‐min intervals. Nonetheless, previous research (Katagiri et al., 2024) has shown that the core temperature threshold and/or the sensitivity of ${\dot{V}}_{E}$ to increasing core temperature are not influenced by prior sodium bicarbonate ingestion.

### 4.7 Conclusion

This study examined the thermoregulatory and chemo‐regulatory responses by manipulating the initial pH to be either mildly acidic or basic. SB supplementation did not decrease absolute ${\dot{V}}_{E}$ during passive hyperthermia but modified ventilatory equivalents, indicating a relative decrease in ${\dot{V}}_{E}$ with regard to CO_2_ removal. In contrast, AC supplementation resulted in higher ${\dot{V}}_{E}$ compared to PLA and SB, likely reflecting respiratory compensation to restore pH. These findings support a role for chemoreceptor activity in modulating hyperthermia‐induced hyperventilation. Thermal discomfort was elevated following both SB and AC supplementation, although the mechanisms remain speculative. Both AC and SB supplementation also tended to shorten immersion time, suggesting a potential reduction in heat tolerance; however, further research is required to investigate if AC and SB supplementation reduce heat tolerance.

## AUTHOR CONTRIBUTIONS

Contributed to the conception and design of the study: Mohammed Ihsan, Jacky Soo, Shernise Ng and Chris Abbiss. Collected the data: Mohammed Ihsan, Jacky Soo and Shernise Ng. Analysed and interpreted the data: Jacky Soo, Mohammed Ihsan and Olivier Girard. Writing the first draft of the manuscript: Jacky Soo and Mohammed Ihsan. All authors reviewed and provided critical feedbacks.

All authors have approved the final version of the manuscript and agree to be accountable for all aspect of the work in ensuring that questions related to the accuracy or integrity of any part of the work are appropriately investigated and resolved. All persons designated as authors qualify for authorship, and all those who qualify for authorship are listed.

## CONFLICT OF INTEREST

The authors have no conflicts of interest, source of funding, or financial ties to disclose and no current or past relationship with companies or manufacturers who could benefit from the results of the present study.

## FUNDING INFORMATION

None.

## Data Availability

The data that support the findings of this study are available from the corresponding author upon reasonable request.
